# Integrating Surface-Based Temperature and Vegetation Abundance Estimates into Land Cover Classifications for Conservation Efforts in Savanna Landscapes

**DOI:** 10.3390/s19163456

**Published:** 2019-08-07

**Authors:** Hannah Victoria Herrero, Jane Southworth, Erin Bunting, Romer Ryan Kohlhaas, Brian Child

**Affiliations:** 1 Department of Geography, University of Tennessee, 1000 Philip Fulmer Way, Room 315, Knoxville, TN 37996-0925, USA; 2 Department of Geography, University of Florida, 3141 Turlington Hall, Gainesville, FL 32611, USA; 3 Department of Geography, Michigan State University, 673 Auditorium Rd., Room 215, East Lansing, MI 48824, USA; 4 Oak Hall School, 8009 SW 14 Ave., Gainesville, FL 32607, USA

**Keywords:** remote sensing, savanna science, NDVI, temperature, MODIS, time series, Landsat, Zambia, protected areas, classifications, South Luangwa National Park

## Abstract

Southern African savannas are an important dryland ecosystem, as they account for up to 54% of the landscape, support a rich variety of biodiversity, and are areas of key landscape change. This paper aims to address the challenges of studying this highly gradient landscape with a grass–shrub–tree continuum. This study takes place in South Luangwa National Park (SLNP) in eastern Zambia. Discretely classifying land cover in savannas is notoriously difficult because vegetation species and structural groups may be very similar, giving off nearly indistinguishable spectral signatures. A support vector machine classification was tested and it produced an accuracy of only 34.48%. Therefore, we took a novel continuous approach in evaluating this change by coupling in situ data with Landsat-level normalized difference vegetation index data (NDVI, as a proxy for vegetation abundance) and blackbody surface temperature (BBST) data into a rule-based classification for November 2015 (wet season) that was 79.31% accurate. The resultant rule-based classification was used to extract mean Moderate Resolution Imaging Spectroradiometer (MODIS) NDVI values by season over time from 2000 to 2016. This showed a distinct separation between each of the classes consistently over time, with woodland having the highest NDVI, followed by shrubland and then grassland, but an overall decrease in NDVI over time in all three classes. These changes may be due to a combination of precipitation, herbivory, fire, and humans. This study highlights the usefulness of a continuous time-series-based approach, which specifically integrates surface temperature and vegetation abundance-based NDVI data into a study of land cover and vegetation health for savanna landscapes, which will be useful for park managers and conservationists globally.

## 1. Introduction

Land cover change is a rapidly growing global concern. In the last century alone, 50% of Earth’s land mass was altered in some way by humans [1]. The rate at which this change is occurring is increasing [2]. We have officially entered the Anthropocene, where human impacts on Earth are so pervasive that we are moving toward “terra incognita”, a world with increased loss of biodiversity, deforestation, and climatic instability [2].

Dryland ecosystems cover approximately 50% of the land surface on Earth [3]. Of these, savannas account for approximately 20% of the global land surface and approximately 55% of the land surface in southern Africa. Savannas are classically defined as a grassland with scattered trees, but in practice they cover a wide variety of covers across the gradient from grassland to denser woodland [3]. Therefore, they are a highly heterogeneous landscape [4,5]. Savannas are such a key ecosystem because they support many human populations and large amounts of floral/faunal biodiversity. They also play an important role in the global carbon cycle and make up almost 14% of global net primary production [6]. These key dryland systems are predicted to be significantly impacted under climate change, which would affect many people, animals, and vegetation [7].

Literature proposes that savannas are patchy mosaics that only exist because there are drivers that prevent them from becoming pure grassland or pure forest [8]. The primary drivers of change in savanna landscapes include changes in precipitation, fire, herbivory, and human pressures, particularly through management, grazing, and agriculture [9,10]. Depending on the specific conditions, these drivers can produce changes in either direction, toward pure grassland or pure forest. Climate, namely precipitation, is one of the most important drivers on this landscape, as drylands are water-limited systems. Precipitation has been found to control the resultant land cover up until a threshold of around 750 mm of total annual precipitation. With precipitation less than this threshold, a grass-dominated landscape is expected. At levels up to 950 mm, a mixed savanna is expected. Above 950 mm, trees become more common and fire plays a bigger regulatory role on the landscape [10]. Above 2000 mm, dense woodlands dominate [11]. Available soil moisture is a measure derived from actual precipitation, temperature, and soil type, among other factors. The Intergovernmental Panel on Climate Change (IPCC) scenarios predict an overall change in precipitation, not in terms of annual amount, but in terms of distribution across the year and increasing variability of precipitation events. This change in distribution, in association with the predicted increased in temperature, would result in a decrease in available soil moisture and therefore may promote shrub growth, because these species tend to be more drought-resistant [12]. Fire helps to maintain the balance between grass and trees and fire frequency is important. There is a relationship between the amount of precipitation and fire. There needs to be enough precipitation that a grass fuel load can build (grass growth), but too much precipitation means that a fire cannot burn [10,11]. With a higher fire frequency, grasslands tend to be dominant and a lower fire frequency tends to lead to a woodier landscape. Landscape management by humans is also a significant driver of change. For example, in Botswana, a fire ban was implemented in the 1990s, which led to an overall lower fire frequency [13]. This resulted in changes in vegetation, including bush encroachment, defined as the increase in woody vegetation [14,15,16]. Herbivory is also an important regulator of vegetation on the landscape. Where there are higher densities of herbivores, there tend to be an increase in bush encroachment, as animals such as elephants destroy trees and grazers prefer to eat grass, thus decreasing competition for shrub species [14].

**Figure 1:**
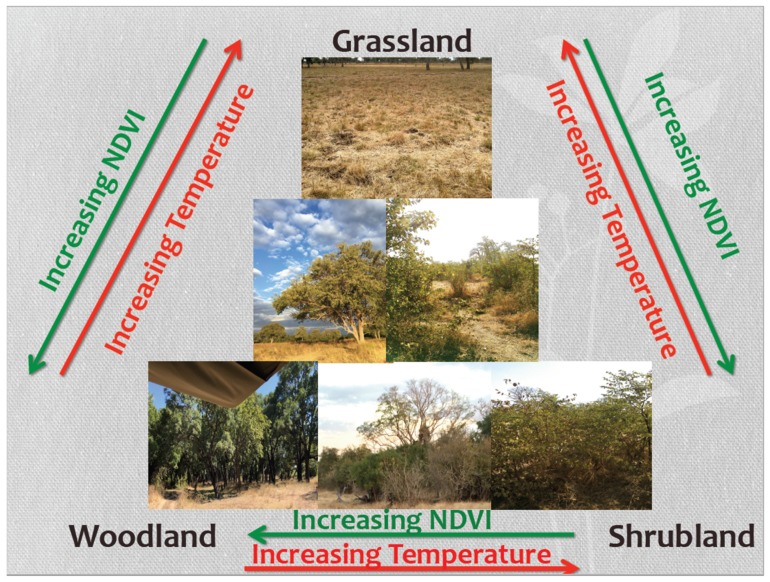
The savanna vegetation continuum represented in terms of surface temperatures and normalized difference vegetation index (NDVI) values for the axes of grassland, shrubland, and woodland cover, with associated field photos from the study site.

Savanna landscapes are key areas of ecological landscape change and yet their heterogeneous structures make them a significant challenge for remote sensing studies. These landscapes represent gradients of grassland, shrubland, and woodland and therefore prove difficult to create meaningful land cover classifications for. A savanna could be any mixture of vegetation structure, which is why certain remote sensing techniques were created as a solution to this complex issue. There are two primary approaches to evaluating savanna systems using remote sensing: Discrete analysis (separate and distinct) and continuous analysis (any infinite interval). Discrete analysis has the advantage of being simple to understand for users on the landscape [16] and the disadvantage that any within-class variation is eliminated [17]. Discrete analysis also tends to be a misrepresentation of how these systems exist, as they exist in gradients, in contrast to more clearly compartmentalized landscapes, for example, agricultural fields. Some discrete measures include land cover classifications [16,18]. Classifications (unsupervised or supervised) attempt to take an image and, based off of a set criterion, break the image pixels into multiple mutually-exclusive classes. However, within savanna landscapes, which present more of a vegetation continuum, these methods tend to result in class separability issues due to the potential spectral confusion between the classes (with shrubland being confused with grassland and woodland). One feasible way for improving land cover characterization is through the application of non-parametric classification algorithms. These types of classifiers are particularly appropriate when the data do not meet the assumptions of parametric algorithms. These assumptions include having normally distributed data, homogeneity of variance, interval data, and independence of data. In these savanna systems, due to their heterogeneity, these land cover classes are often spectrally inseparable because shrub and tree species are often the same, with only a difference in height. Some more statistically robust techniques such as Random Forest and Support Vector Machine have helped to improve this confusion, but still do not perform accurately enough to be usable for managers in these systems [16]. One way to better understand this continuous landscape is to evaluate it by mapping continuous measures, including vegetation indices. However, these continuous measures are still assigned at the pixel level. The normalized difference vegetation index (NDVI) is one such vegetation index. NDVI is a ratio between the near infrared and red bands and can be used as a proxy to model vegetation health and vegetation abundance [19]. NDVI varies between −1 and +1, with larger numbers generally signifying more vegetation. This index saturates out at values around 0.9, but the normal range of NDVI in savannas is 0.2–0.7. In addition, utilizing a time-series approach, incorporating both spatial and temporal (seasonal) variation can also be useful to improve our knowledge of these complex systems [20]. BBST can also be key in separating vegetation cover types [17,21]. The relationship between vegetation amount and temperature is well established, where denser vegetation results in cooler temperatures during the daytime [21]. Thus, in these landscapes, these two continuous measures work most effectively at separating out land cover type when they are coupled together, such as in Lambin and Ehrlich, 1997 and Southworth, 2004 [17,21]. These continuous variables can be measured and when used in conjunction with verified field samples, certain thresholds for vegetation classes can be developed by the user and are therefore more flexible than the traditional discrete classification alone [17,21]. The photographs in Figure 1 depict the gradient and potential confusion between the different land cover classes in these savanna systems. This is the theoretical backbone of this research and further highlights the importance of field-based land cover classifications that can be used to extract longer-term vegetation trends.

This research takes place inside South Luangwa National Park in eastern Zambia. The broad research question is: How has vegetation changed in this eastern Zambian savanna park landscape over time from 2000 to 2016 and what are the best ways of evaluating this change? The goal of this research is to develop a better method of evaluating landscape changes in these sensitive savanna landscapes in order to determine landscape changes in these areas over the last few decades. This research will therefore address the following three questions: (1) How can surface temperature differences across vegetation types be integrated into savanna classification studies to better differentiate the three different savanna class types, i.e., grassland, shrubland, and woodland? (2) How can we use these integrated studies to produce a more accurate land cover classification than traditional classification techniques? and (3) How can this classification be used to detect trends in these land cover classes in the 21st century?

## 2. Materials and Methods

### 2.1. Study Area

**Figure 2:**
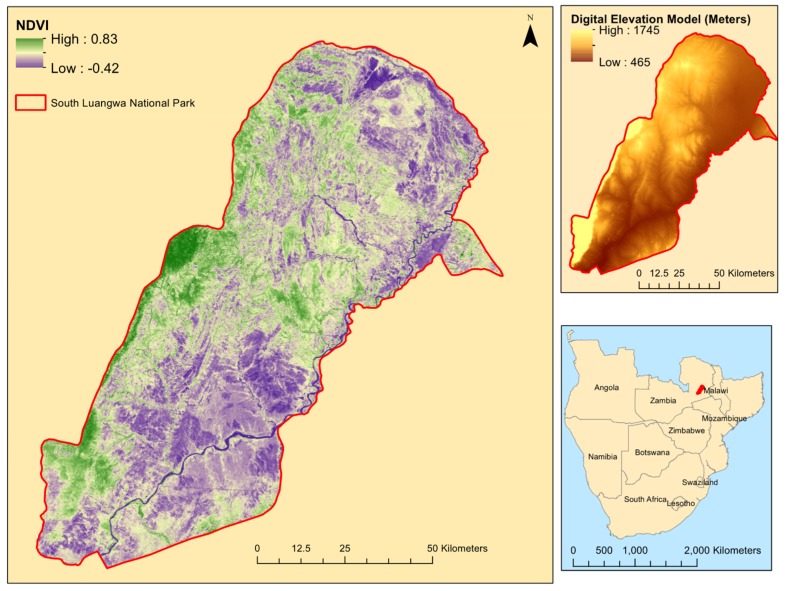
NDVI map of South Luangwa National Park, Zambia from November 2015 (wet season), with a digital elevation model and an inset map.

This study area is South Luangwa National Park, located in eastern Zambia (Figure 2). The geographic location of this park is 12.97 °S and 31.53 °E (WGS84) and it is 9050 km^2^ in area. The average temperature in the park fluctuates between 15 °C in July and 34 °C in November. Over the last 35 years, the mean annual precipitation was 900 mm. There is a gradient of rainfall, with higher precipitation in the west and lower precipitation in the east. Most of the precipitation is delivered during the warm, wet season, which lasts from November to April. The dry, cool season lasts from May to October. The elevation varies from 460 m in the east, rising to a 1750 m escarpment in the west. The Luangwa River is the eastern border of the park and the mountains are the western border. Throughout the park there are some dirt roads, but there are no paved roads within the study area. This is an extremely remote park, with the nearest city, Chipata, over 100 km to the east of the park boundary, but there are a few small towns on the eastern side. The western side is more remote and highly inaccessible, which affects the field sampling accessibility.

Ecologically, this is a savanna park. However, there is a large gradient in the savanna types, with a general trend from east to west of increasing vegetation abundance and highest concentration in the woodlands located in the mountains to the west. Within the central and eastern sections of the park, there is a heterogeneous mix of savannas, including grass savannas, shrub savannas, and wooded savannas. There are also areas dominated by mopane and riparian vegetation is present around streams along the Luangwa River.

The Luangwa Valley has a long history of colonial interactions, including both the Portuguese and the British. In 1898, the British made present-day Chipata the administrative headquarters for Northeastern Rhodesia. In 1900, after the London Convention for the preservation for wild animals, birds, and fish in Africa, regulations on game preservation were issued by the administration in Chipata. Regulation of arms on natives began just after this. The original Luangwa Valley Game Reserve was declared in 1904. In 1913, this decision was reversed and animals responsible for crop damages were shot. In 1925, a Game Ordinance for Zambia was created, but it was not until 27 May 1938, after multiple studies that concluded reserves to be useful for both wildlife and people, that the Luangwa Valley Game Reserve was created again. In the early 1960s Norman Carr began pioneering safaris in the Valley. In 1962, meat production from wildlife began, but in 1964, when Zambia gained its independence, concerns grew over “habitat degradation” and the causes of this degradation. Throughout the 1960s, more safari lodges were built and flights from Lusaka to the Valley were scheduled regularly. From 1967 to 1972, an aid program through the Food and Agriculture Organization, called the Luangwa Valley Conservation and Development Project, was proposed, implemented, and completed. This was the first of many international aid projects that existed over the next decades. Shortly after, management tracks and firebreaks were constructed in the reserve and in 1971 the reserve officially became the national park it is today. The Zambian Wildlife Authority now manages the park and there are ongoing studies on vegetation, wildlife, and socio-economics [22].

This national park contained all of the big five, but from the 1970s through to the 1990s, there was a severe increase in elephant and rhino poaching, with rhinos eventually becoming extinct there. Herbivory is one important driver of vegetation change across the landscape. Herbivory controls vegetation growth at smaller scales and elephants are one of the most impactful herbivores. Elephants ring, debark, and knock over trees [23]. This makes them destructive when densities are too high, but when densities are too low, they cannot help maintain the level of vegetation. Elephant numbers decreased from a peak of 31,000 in 1973 to 9000 in 2013 [22,24,25]. Given this interesting park history, its ecological and socioeconomic importance, and the lack of current research, this park was chosen for analysis.

### 2.2. Remote Sensing Data, Methods, and Inputs

This study strives to integrate multiple continuous datasets to create a more accurate land cover classification of the study area through testing multiple techniques and to further use this classification to extract longer-term ecological trends for this landscape.

#### 2.2.1. Climate Data

Climate is one of the most important drivers in this landscape [26], particularly precipitation, as it controls vegetation growth up to a certain threshold of 750–900 m, and controls growth between grassland, shrubland, and woodland [10]. Higher amounts of rainfall can support greater vegetation growth. Variability in interannual rainfall can also influence vegetation growth across the years, i.e., we expect that even during wet seasons across the years, there may not be the same amount of vegetation growth because it is dependent on the rainfall of that season. Therefore, examination of this variable is critical. Mean annual precipitation was calculated from 1981 to 2016 with the Climate Hazards Group InfraRed Precipitation with Station data (CHIRPS), a gridded rainfall time series dataset [27]. This time period was chosen to determine longer-term environmental change. This dataset had a 0.5° spatial resolution, with a monthly temporal resolution that was aggregated into total annual precipitation across water years (1 October–30 September) [28] for 1981–2016.

#### 2.2.2. Field Data

Field data were collected during the dry season of 2016. Polygons of adequate size, defined as at least 90 m by 90 m areas (or 3 × 3 Landsat pixels), were taken along the dirt roads. Due to dangerous wildlife and access only being via the dirt roads, all samples were taken alongside roads. While this did produce some sampling bias, this was the only option available in this protected area and, given the very low density of traffic and lack of paving, it was determined that the presence of the road had a very limited potential impact. The samples were often extensive in size and so this also minimized any potential impact of road presence in the pixel samples. These samples were taken using a tablet that included a Global Positioning System (GPS) with an accuracy within 4 m. Cover classes identified for classification were grassland, shrubland, and woodland. Grassland cover was defined as a herbaceous dominated area. Shrubland cover was defined as an area dominated by woody vegetation less than 3 m tall. Woodland cover was defined as an area dominated by woody vegetation greater than 3 m tall. These data were collected by determining which of the three classes was dominating any given sample area (80% or higher of the total area sampled). In total, there were 1569 pixels of grassland, 2225 pixels of shrubland, and 3017 pixels woodland used to train the data to account for intraclass variability. The spectral signatures of these points were graphed for the 2015–2016 period to check for outliers and separability across class types. These field data were then used as inputs for classification creation to extract information on NDVI and BBST for each cover type, thus determining possible separability.

#### 2.2.3. Remote Sensing Data Products

1. Landsat 8 Operational Land Imager/Thermal Infrared Sensor (OLI/TIRS) Imagery

The Landsat 8 OLI/TIRS imagery used in this study was pre-processed from the United States Geological Survey (USGS) at two single dates from November 2015 (wet season) and July 2016 (dry season). These two dates were chosen to encompass the seasonal variation of this landscape. This included bands 1 through 12, where bands 10 and 11 were the thermal bands. These bands were all disaggregated to a spatial resolution of 30 m. All of these bands were included individually in our analysis, as well as being input into the data transformations listed below.

2. Data Transformations

There were some important data transformations that were carried out and used as data inputs for the advanced classifier for analysis. The original inputs for these transformations were the Landsat 8 OLI/TIRS imagery from our wet–dry season dates for 2015–2016. One of those transformations is a Tasseled Cap Analysis (TCA). The tasseled cap transformation was performed in the remote sensing analysis software, Environment for Visualizing Images (ENVI). This transformed the data into three bands known as the brightness, greenness, and wetness variables. Another important transformation was the Principle Components Analysis (PCA), which was used to decorrelate the Landsat bands [16]. PCA bands 1, 2, and 3 were the most important in explaining image variance and, in our example, the first three components explained over 95% of the image variance.

3. Landsat 8 OLI/TIRS Normalized Difference Vegetation Index (NDVI)

The normalized difference vegetation index (NDVI) is a ratio between the near infrared and red bands. This is therefore a measure of vegetation greenness and health and can be used as a proxy for vegetation abundance [19]. The NDVI calculated from the Landsat images at a 30 m × 30 m spatial resolution was used as an input for our classifiers.

4. Landsat 8 OLI/TIRS Blackbody Surface Temperature (BBST)

Land surface temperatures proved very useful in a variety of environmental and vegetation studies [28,29,30,31,32,33,34]. The amount of thermal radiation that is emitted at a certain wavelength from an object depends on its temperature and emissivity [35]. In vegetation, the amount of greenness and temperature are inversely related, so greener vegetation, such as a dense forest, has a relatively lower temperature than a barren field, which would have much higher temperatures [17]. Brightness temperature and the spectral radiance are related through Planck’s blackbody equation. The purpose of our use of BBST was to determine a difference in temperatures in our three main land cover classes, i.e., grassland, shrubland, and woodland, without information on land cover. As such, only BBST was derived because, to calculate the actual surface temperature, the surface emissivity (based on land cover) needed to be known. In order to calculate these temperatures, the first step was to convert the Landsat imagery data from radiance to reflectance. This was done through a series of equations, beginning with:

Lλ = ((LMAX − LMIN/QCALMAX − QCALMIN)) * (DN − QCALMIN) + LMIN,

where DN is the quantified calibrated pixel value, LMIN is the spectral radiance scaled to QCALMIN, LMAX is the spectral radiance scaled to QCALMAX, QCALMIN is the minimum quantified calibrated pixel value (LMIN) in DN, and QCALMAX is the maximum quantified calibrated pixel value (LMAX) in DN. The above-defined constants could be found in the metadata of the imagery. Then, Lλ was used as an input in the temperature equation, where k_1_ and k_2_ are prelaunch calibration constants specific to the satellite:

T = (k_2_)/(ln((k_1_/Lλ) + 1)).

Then, the data needed to be converted from Kelvin into Celsius using (−273.15°). This produced a map of temperatures across the landscape and our field samples of different land cover types were overlaid for comparison and extraction.

BBST was created with the thermal bands (10 and 11) of the Landsat 8 OLI/TIRS imagery for the November 2015 wet season and July 2016 dry season images. Mean BBST data values were extracted for each of the three land cover types using our field-verified data samples. These mean values for BBST were then graphed for comparison and separability between land cover types. Eventually, rules for our rule-based classifier were developed using these data values.

5. Moderate Resolution Imaging Spectroradiometer (MODIS) Normalized Difference Vegetation Index (NDVI)

MODIS MOD13Q1 data from the United States Geological Survey (USGS) was used in this paper. This was a 16-day maximum NDVI value by pixel product at the 250 m spatial resolution [28]. Our dataset was created by taking this maximum value product and averaging these by season (March, April, May; June, July, August; September, October, November; December, January, February) for each year from 2000 to 2016. We used the full extent of the available data once we established our cover types in order to look at the longer-term changes in vegetative cover across this landscape; therefore, we utilized the full extent of the MODIS time series, within which our field data collection points were embedded. Therefore, 2000–2016 was chosen as the time period, because this was the period of available MODIS data. The month groupings were chosen based on climate regime. Averaging the maximum NDVI product allowed for minimization of missing data and the use of only high quality pixels. This MOD13Q1-based NDVI time series was used to incorporate vegetation abundance trends over the time period of 2000–2016, thus determining any potential changes within each cover type across the sixteen-year time series.

### 2.3. End-Product Analysis

#### 2.3.1. Land Cover Classifications

Land cover classifications are a discrete analysis that describe a landscape and its land cover types. With these discrete end-product maps, interpreting the geometry and arrangement of classes is conceptually more simplified [17], such that stakeholders may have a quantifiable map of change over time.

**Figure 3:**
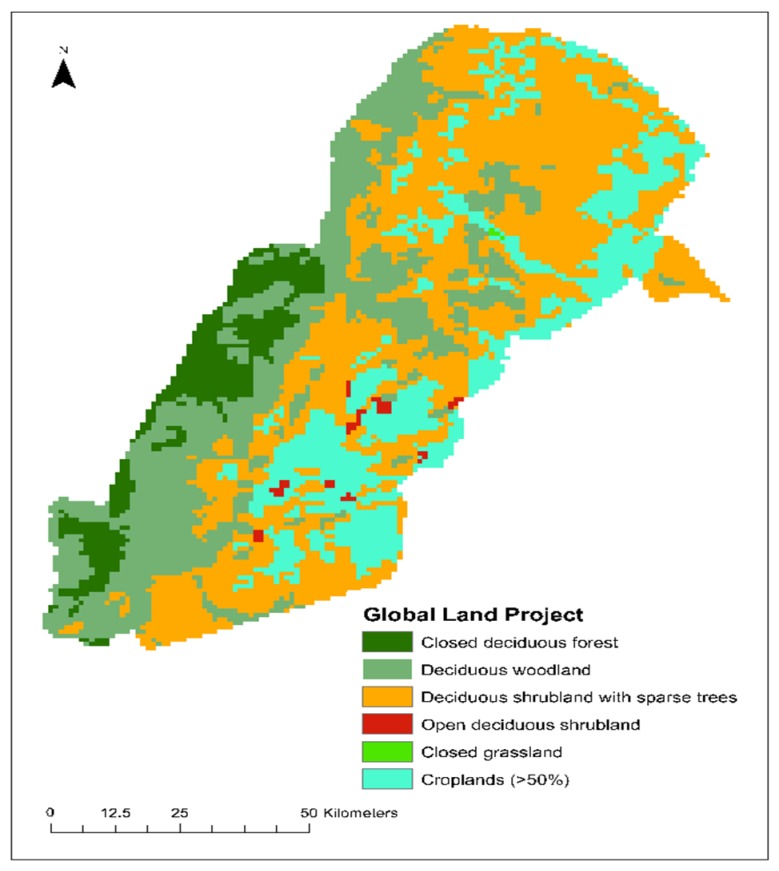
Global Land Project Classification of South Luangwa National Park. Source: Global Land Project, 2016.

Refined classifications on these complex landscapes are needed because global-scale categorical maps fail to recognize the nuances in this landscape, such as the Global Land Project Classification. According to this classification, 20% of the protected area in our study area is classified as crop, which is inaccurate, as there is no crop area within the park (Figure 3). This emphasizes the importance of alternative approaches to more accurately represent these heterogeneous landscapes. This research presents an innovative way to determine these grassland, shrubland, and woodland continuums using continuous metrics in a landscape in eastern Zambia and then puts this into a larger context of regional vegetation and climate dynamics.

One type of non-parametric classifier is Support Vector Machine (SVM). This classifier is a supervised learning algorithm, which uses statistical learning theory to make predictions about land cover types. SVM is efficient at separating noisy data through pattern recognition [36]. The SVM classification was completed in the remote sensing processing software, ENVI, using our training sample polygons. This classification was performed on the water year (wet and dry season) images using Landsat 8 OLI/TIRS bands 1–12 for the November 2015 and July 2016 (wet and dry seasons) images as inputs and derived Landsat NDVI, Principle Components Analysis (PCA) PC 1–3, and the three Tasseled Cap Analysis (TCA) outputs.

One way to integrate intensive field-based data in order to make more accurate products is through the use of rule-based classifications. This classification functions as a series of IF–THEN statements, where IF is the condition and THEN is the conclusion [37]. These rules need to be developed based off of individual images. In a rule-based classification, the user sets the parameters and can include any variables. In this study, the rules for this classification were developed from the November 2015 wet season imagery NDVI and BBST coverage. This was because these NDVI and BBST datasets had the largest separability between land cover classes, as determined by extracting the values for these datasets across our training sample data. This classification technique led to more specific and customized rules for each individual image classified, with potentially more accurate results of change over time.

#### 2.3.2. Mean MODIS NDVI Time Series 

By using our training field data, we were able to create a highly accurate, rule-based land cover classification using only one wet season image at the Landsat spatial resolution and then used this classification to extract mean NDVI values over time from MODIS. This mean NDVI time series was created by coupling the rule-based classification from November 2015 with the previously described MODIS NDVI data. In order to address the difference in scale between Landsat and MODIS, an aggregated polygon was created from each of the classes in the classifier. Zonal statistics were then performed on the MODIS NDVI imagery and values were extracted by land cover class. This resulted in a mean NDVI of the woodland, grassland, and shrubland by season over time. 

## 3. Results

### 3.1. Climate Data

**Figure 4:**
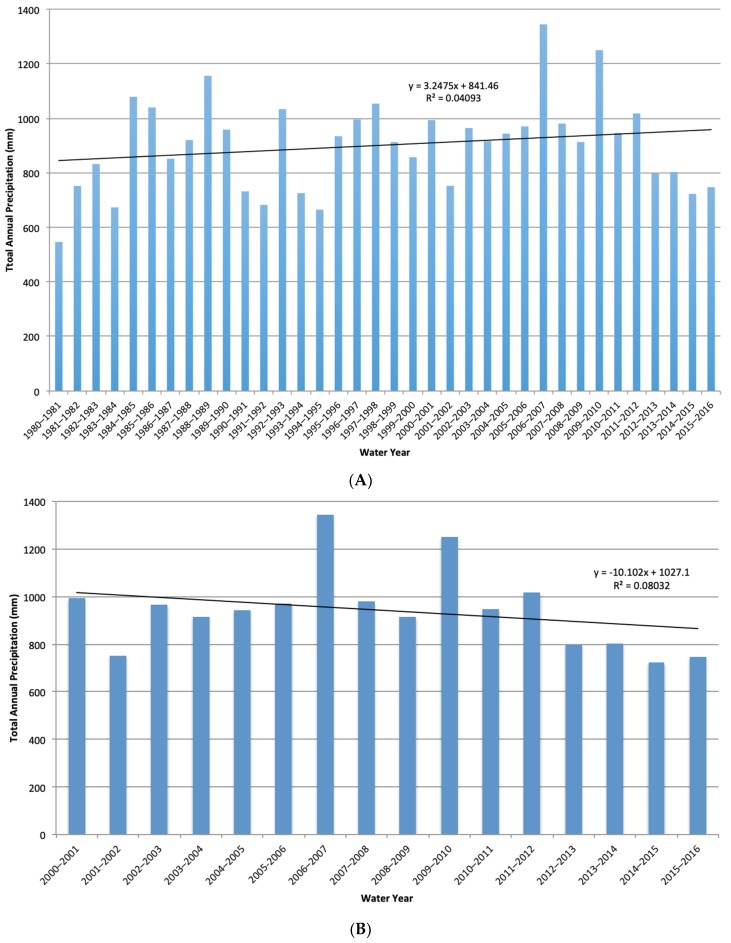
Precipitation for South Luangwa National Park, Zambia for (**A**) total annual precipitation from 1980 to 2016 with a linear trend line and (**B**) total annual precipitation from 2000 to 2016 with a linear trend line.

Over the last thirty-five years from 1981 to 2016, in South Luangwa National Park, the mean annual precipitation was 900 mm (Figure 4). This long-term data was plotted to determine the general climate of this park. From this, the 21st century data were extracted for closer examination to match our study dates. There was interannual variation in this time series from 1980 to 2016, with an overall positive trend over time. However, from 2000 to 2016, there was a decline in precipitation, although this decrease was not statistically significant. Decreases in precipitation may however lead to a decrease in vegetation growth and productivity, as measured through NDVI.

### 3.2. Landsat NDVI Analysis

When NDVI values were extracted as a polygon mean for each of our training sample areas, grassland, shrubland, and woodland, there was some separability. During the wet season, woodland always had the highest NDVI value, averaging between 0.5 and 0.7. There was confusion in separability between grassland and shrubland across seasons, with values varying between grassland (0.3–0.6) and shrubland (0.3–0.5). There was more confusion in the results during the dry season, with all of the classes having similar values (0.2–0.35). Therefore, NDVI should not be the only measure for class distinction. From this, NDVI values were extracted at the pixel level in the training sample polygons for analysis with the November 2015 imagery.

### 3.3. Landsat BBST Analysis

In the wet season, there was an overall higher BBST than in the dry season. There was some variability between classes as well, with woodland always being the coolest class across the seasons (32 °C in November and 23 °C in July). Grass and shrub were more similar in November, both having a temperature of 36 °C, and only slightly more separable in July, with grass having a slightly cooler temperature of 26.8 °C and shrub having a slightly warmer temperature of 27.2 °C. Therefore, temperature is useful for the separation of woodland, but there is still confusion between grass and shrub, so this measure should not be used alone. From this, BBST values were extracted at the pixel level in the training sample polygons for analysis with the November 2015 imagery.

### 3.4. NDVI versus BBST

**Figure 5:**
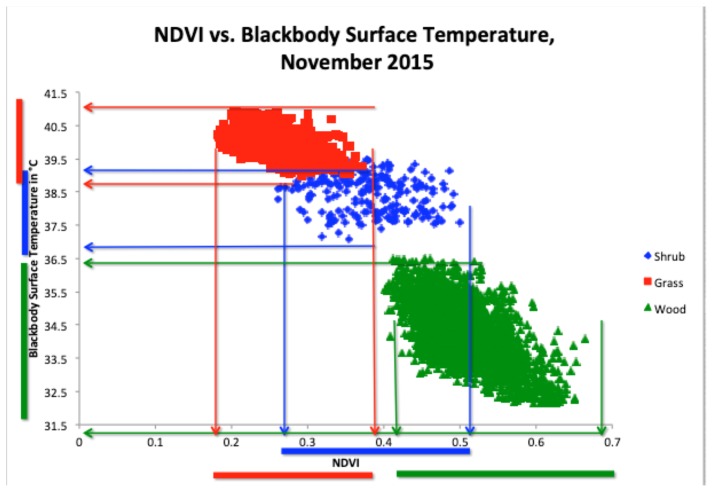
NDVI plotted against blackbody surface temperature (BBST) to distinguish land cover types in November (wet season) 2015 in South Luangwa National Park.

Given the confusion between grass and shrub with each of these variables individually, we used the novel approach of using them together to tease out interclass separability (Figure 5). This was a by-pixel analysis for each of the training sample polygons. There was separability in mean NDVI when coupled with BBST in our three covers, with woodland cover having the highest NDVI and lowest BBST, shrubland cover having the middle NDVI and middle BBST, and grassland having the lowest NDVI and highest BBST.

In the November (wet) 2015 image data, when NDVI (x-axis) and BBST (y-axis) were plotted against each other, woodland separated out very well, with the highest NDVI values ranging from 0.4–0.68 and the coolest temperatures ranging between 32.5 °C and 36.4 °C. Grass separated out with the overall lowest NDVI values, between 0.18 and 0.38, and the overall highest temperatures, between 38.6 °C and 41.3 °C. Shrub was in the middle of the graph with overlapping NDVI ranging from 0.28 to 0.51 and overlapping temperatures ranging from 36.7 °C to 39.4 °C. Without plotting these two variables together, grass and shrub would not be clearly separable. The results the boundaries between which NDVI and temperature fell were used to develop an accurate rule-based classification.

### 3.5. Support Vector Machine Classification

**Figure 6:**
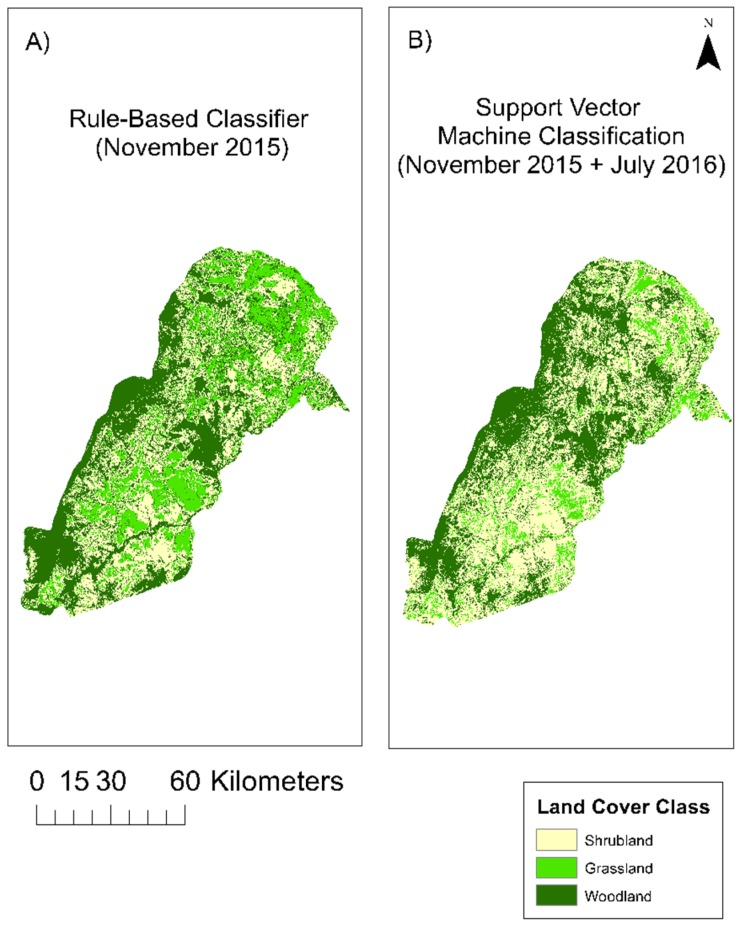
Classification results for (**A**) rule-based classification for November (wet season) 2015 and (**B**) the Support Vector Machine classification for November (wet season) 2015 and July (dry season) 2016, in South Luangwa National Park, Zambia.

The SVM classification was performed using data from November 2015 (wet season) and July 2016 (dry season) together to increase classification performance (Figure 6). The percentages of class type were calculated. Higher vegetation abundance (woodland) was found at higher elevations and around rivers and represented 40.02% of the landscape. Grassland was found more often near floodplains and at lower elevations and represented 10.51% of the landscape. Shrubland was in mixed areas and represented 49.47% of the landscape. The overall accuracy of this classification was 34.48% (Table 1) using randomly selected testing points, which were excluded from analysis.

### 3.6. Rule-Based Classification

For the rule-based classifier, rules were developed from the field data and linked to the images (Figure 6). The final set of rules created for the November (wet season) 2015 image were: (1) IF NDVI was higher than 0.41 and BBST was less than 36.5 °C, THEN it was the wood class. (2) IF NDVI was lower than 0.36 and BBST was greater than 38.6 °C, THEN it was the grass class. (3) IF NDVI was between 0.28 and 0.51 and BBST was between 36.7 °C and 39.3 °C, THEN it was the shrub class. Woodland represented 41.20% of the landscape, grassland represented 22.87%, and shrubland represented 35.93% of the landscape. This classification had a higher overall accuracy of 79.31% (Table 1). This classification accuracy was established via the development of an error matrix based on known cover types, which were held back from the initial classifications. As such, the rule-based classifier significantly outperformed the support machine vector classifier, as illustrated in Table 1. The main limitation of such rule-based classifiers is the need to develop rules for each image date, which can be very time-consuming, and, if requiring fieldwork, can also be expensive. Once developed, however, such products can be utilized in additional analyses where differentiation of cover type is a necessary first step. This was the case in this research where, based on this much more accurate rule-based classification, we were able to utilize this classification product in the longer-term MODIS-based time series analysis to determine longer term vegetation change across this landscape.

### 3.7. Support Vector Machine versus Rule-Based Classification

Even though there was a great degree of variation of accuracy between these classifiers, some insight may be provided about land cover class percentage, distribution, and confusion. There were some differences in percentage within classes across these classifications. With the rule-based classifier (RBC), the landscape was 35.93% shrubland, which is 13.54% less than the SVM. In the RBC, 22.87% was grassland, which is 12.36% more than the SVM. In the RBC, 41.20% was woodland, which is 1.18% more than the SVM, so they were very similar. The RBC had an accuracy of 79.31%, which was 44.83% more than the SVM, making it much more accurate. When evaluating the classified maps, the issues of class confusion were highlighted. In Figure 6, which is a comparison of the resultant maps from the two different classifiers, the maps suggest that the confusion in both of the classifiers was within the grass and shrub classes. Given this higher accuracy of the RBC, this suggests that the SVM was under predicting grass. However, there were some similar spatial distributions of classes, with woodland occurring in higher elevation areas and around streams, while grassland occurred at lower elevation and floodplains and shrubland was a mix of the two.

### 3.8. Mean MODIS NDVI Time Series

**Figure 7:**
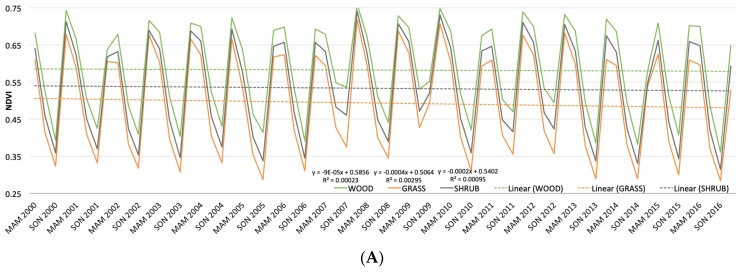

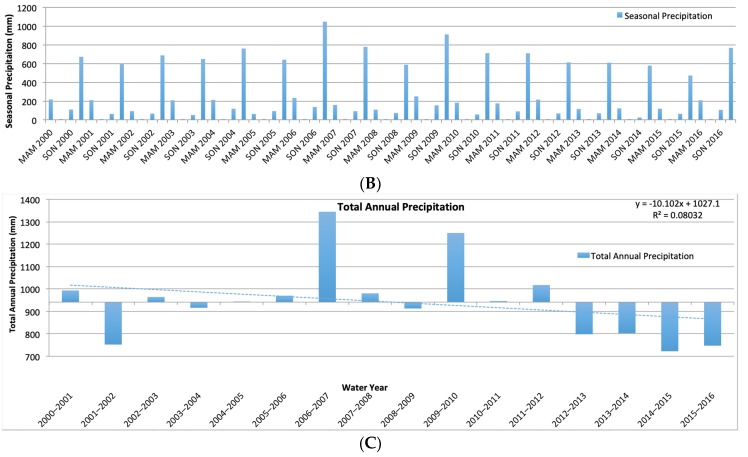
(**A**) Mean NDVI by land cover class (woodland, grassland, and shrubland) by season (MAM: March, April, May; JJA: June, July, August; SON: September, October, November; DJF: December, January, February) from 2000 to 2016. (**B**) Total seasonal precipitation (MAM, JJA, SON, DFJ) from 2000 to 2016. (**C**) Total annual water year precipitation from 2000 to 2016, where the horizontal axis represents the mean annual precipitation over this time period (941 mm).

These classes separated out very clearly across all seasons and time periods (Figure 7A). Woodland always had the highest NDVI, followed by shrubland in the middle, while grassland always had the lowest mean NDVI. We expected woodland and shrubland values to be more similar because there were some overlapping species across these classes, with only a difference in height and abundance. Therefore, because these two classes were highly separable in this time series, it supported the usefulness of our classification technique. In the woodland class, there was a slight decrease in NDVI over time, whereas there was a slightly greater decrease in shrubland NDVI and a more significant decrease in grassland NDVI over this time period. Overall, while the patterns were highly variable seasonally and year-to-year, there were clear gradual declines in the amount of vegetation cover, especially grassland, over the study period.

Given that precipitation is one of the main drivers in dryland landscapes, we compared trends in NDVI values by season and over time to trends in precipitation values. In Figure 7B, total seasonal precipitation (MAM, JJA, SON, DJF) was represented annually over the time period of 2000–2016. In MAM, SON, and DJF, there was a decreasing trend in precipitation over time. JJA (dry season) had a negligible amount of precipitation. Figure 7C shows the total annual precipitation over the water years from 2000 to 2016, with a mean annual precipitation of 941 mm as the horizontal axis. Columns above the horizontal axis indicate years with higher than average precipitation (wetter) and columns below the horizontal axis indicate years with lower than average precipitation (drier). Overall, there was a marked decrease in total annual precipitation, indicated by the trend line, with values at the end of the time period below the mean annual precipitation.

The decreases in precipitation did appear to match the declines in NDVI, although variations existed based on land cover class. Overall, the grasslands were more significantly impacted and were a vegetation class that responded much more quickly to changes in precipitation, due in part to shallower root systems. Shrubland also showed a decrease, although not as significant as woodland, again reflecting the time it takes for these cover types to respond to changes in precipitation. Even with this decrease in precipitation reflected in the vegetation covers, there was significant seasonal variation and, most likely, real time lags between changes in precipitation and resultant changes across landscapes. Even looking across seasons for NDVI, some of these changes were probably still obscured. In addition, our series was only sixteen years long, which is a fairly short time to see changes in resultant vegetation, especially for gradual changes in precipitation such as those illustrated here. For example, examining these two datasets together, the lowest NDVI values of the year (JJA-dry season) were not as low when there had been more precipitation in the preceding season (MMA-wet season). Our results suggest that the timing of precipitation during a season may also be an important factor and worthy of more study, especially with intra-annual changes in precipitation likely occurring more frequently under a changing climate. In the final few years of this time series, precipitation amounts of below 750 mm per annum were recorded (from 2013 through 2016). This value is one often highlighted in the savanna literature as determining a more wooded versus grass-based system and so may represent a significant threshold for this landscape. If future precipitation trends continue to be below 750 mm per annum, then this may have more drastic consequences for vegetation cover across this region and shifts to more grassland-dominated states may occur. Future research should continue with this analysis thread, as a longer time-series is needed and future climate changes are still uncertain across this region. Clearly, however, continued monitoring is essential both from the climate and vegetation cover standpoints.

## 4. Discussion

The necessity for discrete maps for managers to be able to quantify change on their landscapes continues, even though significant detail is lost with discrete data [17]. However, given the difficulties in using remote sensing techniques in this highly heterogeneous landscape, more advanced techniques need to be developed in order to effectively create these discrete products [16,17,18]. Our intensive field data, coupled with continuous measures, i.e., NDVI and BBST, creates a highly accurate, rule-based classification. The novel use of this classification as an input for the MODIS NDVI time series allows us to better understand vegetation cover in such a dynamic system and is therefore of significant use to park managers and conservationists globally. While the initial development of the rule-based classification is location- and time-dependent, once developed, it can be quickly applied to the larger landscape and changes over space and time can be addressed. This two-fold analysis, with the development of more accurate products for the differentiation of savanna types and application to MODIS time-series data, is a relatively fast and easy tool for managers and conservationists to adopt.

This paper contributes to the broader literature of savanna remote sensing and degradation in savannas. By using our highly accurate, rule-based classification as an input for the MODIS NDVI time series data from 2000 to 2016, we can see that vegetation cover in this landscape has indeed declined in abundance over time, with more significant declines in grassland compared to shrubland and woodland (Figure 7A). We might expect this given the similarity in woody species between woodland and shrubland. There may be biological implications of a decline in grassland health on the landscape, because many grazing species depend on this grass. Other studies have also shown the importance of maintaining grass swards for maintaining biodiversity in grazers [16,38]. This decline in vegetation productivity may also lead to socioeconomic implications, as tourism depends on wildlife abundance and diversity and communities depend on this ecotourism.

Given the improvement in classification of these field data, this study highlights the importance of field data collection. Data collected from the field in the form of land cover, which were utilized to develop discrete classes of woodland, shrubland, and grassland, were valuable in additional time-series-based analyses. This field data, when extracted at the pixel level, helped to separate out class types in order to develop rules for a rule-based classifier. Therefore, this study also highlights the usefulness of coupling continuous data with discrete methods to create a stronger product for quantifying vegetation. For this technique, we only needed one image date (November 2015—wet season) in order to create a much more accurate classification, with an overall accuracy of 79.31%. The use of this single-date classification as a basis for a time-series-based MODIS NDVI analysis from 2000 to 2016 also showed how products can be developed from field-based analyses and applied to much longer spatial and temporal scales to allow us to better understand the longer-term dynamics that impact these parks. Undertaking the rule-based classification each year would be too expensive and labor intensive, therefore its use as part of a larger analysis is more ideal and much more manageable for park managers.

The Support Vector Machine (SVM) classifier, an advanced algorithm, is based off of using machine learning alone, therefore it may have continuous difficulty separating out these classes. While the SVM also had some continuous inputs, such as NDVI, this method did not use the more human-driven technique of creating classification rules, making it less accurate in this heterogeneous landscape. The SVM required multiple images during the water year (from both the wet and dry season) in order to make the products, which ended up with a very low level of accuracy. Relative to the rule-based classification, the SVM under-predicted the grassland class, with probable confusion between shrubland and grassland because the woodland classes were of similar proportions and distributions in both classifications. The overall accuracy of the SVM was 34.48% for the 2015–2016 water year. This is also an important finding, as the assumption that more complex and advanced classification methods result in higher accuracy is untrue for this landscape. Instead, the two simple coverages, NDVI and BBST, were much more superior differentiators of class type and were much less computer intensive and easy to run. These findings are important and especially key for resource-limited and often technology-limited regions of the world.

The main drivers on these savanna landscapes are precipitation, fire, herbivory, and humans. This slight decline in NDVI does appear to be linked to the decrease we see in precipitation on the landscape (Figure 4 and Figure 7), especially since 2006. However, NDVI may continue to decline more drastically under new regimes brought on by climate change [9,10]. This may also have biological implications for the future, as a decrease in precipitation was shown to promote bush encroachment, which may be defined as an increase in lower-quality shrub species [14,15], although we are currently seeing a decline across all classes. Although human management has stayed consistent over this time period, natural fires may be increasing due to the decline in precipitation, which would promote grass layers [13,39]. However, if there is not enough precipitation to produce the necessary fuel load in grass, we may not see an increase in fires [10,11]. This is one potential implication of a decrease in NDVI across these classes. The other major driver is herbivory. One of the landscape engineers is the elephant and we have seen their numbers drastically decrease in the Luangwa Valley over time, from about 30,000 in 1975 to 9000 in 2015 [22,24,25]. Some studies suggested that this decrease in large herbivores may lead to an increase in bush encroachment [14]. This may also promote growth of tall trees, as has been found in other studies [40]. However, despite this decrease in herbivores in the park, we saw an equal decline across all three classes, so herbivory is likely not a major driver here. This is possibly because, unlike some other parks in southern Africa, such as Chobe National Park in Botswana [16], elephant densities have consistently been relatively low.

## 5. Conclusions

For this study, a field-supported, rule-based classification was necessary because more traditional classifiers are not very accurate in this landscape. The Support Vector Machine technique was only 34.48% accurate due to the landscape heterogeneity and confusion between classes (due to the same species being present in both). Using intensive field data, we were able to separate out land cover types using continuous NDVI and BBST data. This data was then used as an input into a much more accurate rule-based classification (79.31% accuracy). This rule-based classification was used to extract MODIS NDVI values in a time series from 2000 to 2016. There was a decline of vegetation abundance across all vegetation cover types within this park, especially across grassland, during this time period. This decline does appear to be linked to the decline in precipitation over this same time period, although continued monitoring is necessary to better establish this and to determine if this decline in both precipitation and vegetation abundance continues.

This study highlights the usefulness of a single-date classification, which explicitly integrates surface temperature and vegetation abundance data into a study of land cover and applies the findings on a larger spatial and temporal scale using MODIS data, specifically, the integration of field-based data products for a single point in time, to better define complex savanna landscapes and to use these data as inputs for the separation of savanna types for a time-series-based analysis of vegetation change over time. Managers should continue to monitor vegetation on the ground in these highly heterogeneous and sensitive landscapes, as they are important regions for biodiversity, conservation, science, and socioeconomics, but should also readily incorporate these field-based studies into remotely sensed, longer-term, time-series-based analyses. These integrations across scales and remote sensing platforms will prove to be an excellent tool for managers and conservationists globally.

## Figures and Tables

**Table 1:** Error matrix for the rule-based classification and the Support Vector Machine classification for the 2015–2016 water year in South Luangwa National Park.

Error Matrix	Woodland	Shrubland	Grassland	Total	Class Error Omission %	Class Error Commission %
**Rule-Based Classification**						
Woodland	23	8	1	32	20.69	28.13
Shrubland	6	25	3	34	24.24	26.47
Grassland	0	0	21	21	16.00	0.00
Total	29	33	25	174	-	-
**Support Vector Machine**						
Woodland	18	13	1	32	60.87	43.75
Shrubland	19	11	4	34	62.86	44.11
Grassland	9	11	1	21	83.33	95.00
Total	46	35	6	174	-	-

Rule-based classifier (RBC) overall accuracy: 79.31%; Support Vector Machine (SVM) overall accuracy: 34.48%.
